# A systematic literature review on analytical thinking development in mathematics education: trends across time and countries

**DOI:** 10.3389/fpsyg.2025.1523836

**Published:** 2025-06-26

**Authors:** Mingda Wang, Mohd Effendi Ewan Mohd Matore, Roslinda Rosli

**Affiliations:** ^1^Faculty of Education, The National University of Malaysia (UKM), Bangi, Malaysia; ^2^Research Centre of Education Leadership and Policy, Faculty of Education, The National University of Malaysia (UKM), Bangi, Malaysia; ^3^STEM Enculturation Research Center, Faculty of Education, The National University of Malaysia (UKM), Bangi, Malaysia

**Keywords:** analytical thinking, mathematics education, systematic literature review, cognitive process, teaching strategies

## Abstract

**Introduction:**

Analytical thinking, which involves deeply understanding and dissecting information, is crucial in today’s data-rich society. In education, it develops students’ ability to think deeply, deconstruct problems, and evaluate evidence to enhance academic performance and critical thinking skills. However, past research has not thoroughly examined the patterns of analytical thinking studies, particularly in terms of year and country classifications, which are essential for identifying research trends and gaps.

**Methods:**

Hence, this study aims to classify studies on analytical thinking by year and country using Systematic Literature Review, because this data is useful for understanding research trends. By using a methodology according to PRISMA, the research process was divided into four key stages: searching, screening, analyzing, and summarizing. A total of 21 relevant articles were carefully selected and analyzed in depth through targeted searches on main databases such as Web of Science, Scopus, and Google Scholar.

**Results:**

The main findings reveal a clear trend of increasing research on analytical thinking in mathematics, with notable peaks in 2021, and Indonesia emerging as the leading contributor in this area.

**Discussion:**

This finding has important implications for shaping educational policies and curriculum development, particularly in countries aiming to foster analytical thinking skills in students. Future research should broaden the scope by incorporating more disciplines and diverse theoretical frameworks, enhancing the application and impact of analytical thinking across fields.

## 1 Introduction

The innovation of Internet technology has led to the widespread application of Big Data across various industries and fields, significantly shaping people’s knowledge systems and lifestyles (Özdemir and Hekim, 2018). The advent of the Big Data era means an explosion of information, and analytical thinking enables people to extract key insights from massive amounts of data to support data-driven decision-making (Sarker, 2021). This way of thinking also has a positive impact on innovation, scientific research and career competitiveness, helping to solve global challenges and improve media and information literacy (Meissner and Shmatko, 2019; Walker et al., 2022).

Analytical thinking is not only a competitive advantage for individuals and organizations but also an indispensable competency that drives social progress and sustainable development (Güner and Erbay, 2021). It is a complex process that aids in understanding intricate problems, making informed decisions, and advancing scientific research and innovation (Graesser et al., 2018). This critical thinking skill involves deep thinking, comprehending information, breaking down problems, evaluating evidence, and generating reasoned ideas and conclusions (Chang et al., 2022). As society faces complex and ever-changing challenges, analytical thinking helps in developing a deeper understanding of these problems, thus supporting the development of effective solutions (Maani and Shanti, 2023). Therefore, fostering and promoting analytical thinking is crucial to better address the complexities and challenges of modern society (Coulson and Thomson, 2006).

Analytical thinking is different from synthetical thinking and creative thinking and is a part of systemic thinking and critical thinking. The difference between analytical thinking and synthetical thinking is decomposition and aggregation, one is identifying differences and the other is finding similarities and the other is finding similarities. Creative thinking is relating or creating previously unrelated things or ideas, while analytical thinking contributes to creativity (Cromwell et al., 2023). Systemic thinking is a simple thinking technique for gaining systemic insight into complex situations and problems (Nguyen et al., 2023). Its basic idea is to list more different elements, which is consistent with analytical thinking. Regarding critical thinking, its definition is complex and esoteric and has changed in recent years, and some of the definitions of critical thinking include analytical thinking (Lau, 2024). Facione (2015) mentioned that critical thinking is the ability to interpret, analyze, evaluate, infer, explain, and self-regulate when dealing with various types of information and situations. Paul and Elder (2013) described it as the disciplined art of ensuring that you use the best thinking you are capable of in any set of circumstances. Halpern (2013) emphasized that critical thinking involves the use of cognitive skills and strategies that increase the probability of a desirable outcome, such as analyzing facts, generating and organizing ideas, defending opinions, making comparisons, drawing inferences, evaluating arguments, and solving problems. Ennis (2018) noted that it involves reflective and reasonable thinking focused on deciding what to believe or do.

Analytical thinking is a crucial tool for understanding complex situations, particularly in mathematics education, where it involves two main abilities: breaking down mathematical problems to assess their components and applying deep analysis to find insightful solutions. This approach is essential for developing the key skills needed to understand and solve mathematical problems effectively. Dwyer et al. (2014) proposed a framework for critical thinking that includes cognitive competencies like interpretation and analysis, as well as reflective dispositions. Abrami et al. (2015) introduced a “Four-Dimensional Model” for critical thinking, which emphasizes explanation, evaluation, inference, and decision-making as key components. This model highlights the ability to scrutinize arguments and recognize logical errors, assess evidence validity, and identify inconsistencies. Some research focuses on individual differences in analytical thinking. Toplak et al. (2014) explored cognitive abilities and biases in reasoning, while Pennycook and Rand (2020b) examined how people often overestimate their analytical thinking abilities. Baron (2012) discussed the role of analytical thinking in decision-making and problem-solving.

Analytical thinking is valuable in academia, education, business, and daily life. It aids decision-making (Sofi et al., 2023) by helping individuals make informed choices, and in problem-solving (Almulla and Al-Rahmi, 2023) by breaking down complex issues. Additionally, it drives innovation and creativity, fostering connections across different fields. In education, analytical thinking is essential for developing problem-solving skills, promoting independent learning, and strengthening critical thinking (Nilimaa, 2023; Jamil et al., 2024). It also plays a key role in scientific research, helping students design experiments, analyze data, and formulate hypotheses (Marnı et al., 2020). Furthermore, it encourages interdisciplinary thinking and helps students connect knowledge across various disciplines (Gardiner, 2020).

In mathematics education context, it is not only helping students to understand mathematical concepts more deeply, but also develops the key skills they need when solving mathematical problems. Through analytical thinking, students are able to break down complex problems into smaller parts, reason logically, prove theorems, think abstractly, and apply mathematics to real-life problems in modelling and data analysis. In addition, analytical thinking stimulates students’ spirit of exploration and discovery, enabling them to ask questions and discover mathematical patterns on their own (Kholid et al., 2020). Analytical thinking prepares students for their future careers, as many careers require analytical thinking skills (Rios et al., 2020). Ultimately, analytical thinking encourages innovation and creativity and develops students’ ability to think of novel solutions (AlAli, 2024).

Although the importance of analytical thinking is well recognized, current research has some shortcomings. Firstly, education systems often emphasize memorization and standardized tests over analytical thinking development (Dorji, 2023), resulting in students excelling in knowledge but lacking critical thinking skills (Fischer et al., 2020). Secondly, measuring analytical thinking remains challenging due to inconsistent standards. Additionally, cultural and social differences affect the development of analytical thinking, necessitating further research to find universally applicable methods (Paul and Elder, 2006; Sternberg and Halpern, 2020). Furthermore, as technology advances, the risk of information overload and misinformation increases, highlighting the need for better analytical skills in the digital age (Pennycook and Rand, 2020a).

For these reasons, conducting a Systematic Literature Review (SLR) focused on the year and country of studies related to analytical thinking is essential. By analyzing research trends across different years and countrys, the SLR can identify gaps in how analytical thinking is emphasized or overlooked in various educational systems. It can also reveal patterns in the development of analytical thinking, shedding light on the impact of cultural, social, and technological factors. This comprehensive review will help establish more consistent standards and guide future research toward developing universally applicable methods to improve analytical thinking skills globally.

The motivation for conducting this SLR stems from the need to address the gaps in the current understanding of analytical thinking’s development and measurement. This study is important because it systematically classifies existing research by year and country, providing insights into global trends and areas needing further exploration. By identifying these patterns, the study benefits educators, policymakers, and researchers by highlighting effective strategies and areas where analytical thinking is underdeveloped. This is crucial for advancing knowledge, as previous research has often lacked a comprehensive overview of how analytical thinking is approached worldwide. Understanding these classifications will help tailor educational strategies to different cultural contexts and improve analytical thinking skills globally.

In order to enhance the understanding and development of analytical thinking in mathematics education, it is essential to examine the distribution and focus of existing literature. Therefore, this study aims to classify prior research on analytical thinking based on publication year and country. Specifically, the study has two objectives: (1) to analyze publication trends over time to identify growth patterns, and (2) to examine the country distribution of studies across various nations, such as Indonesia, the United States, and Malaysia, to gain insights into the global landscape of research. By addressing these trends and gaps, the study seeks to inform and refine educational strategies and policies, ensuring that analytical thinking in mathematics is effectively promoted across diverse cultural contexts.

## 2 Methods

A Systematic Literature Review (SLR) is a research methodology used to collect, assess and summarise existing literature on a specific topic or research question in a comprehensive and organised manner. Systematic reviews are widely utilized in scientific research, academic studies, and policy development to offer comprehensive and current summaries of knowledge (Page et al., 2021). In this study, all articles should be related to “analytical thinking,” and the main goal is to conduct a comprehensive search for academic research on analytical thinking, especially for mathematics students, in order to make a compendium for subsequent research. Following the PRISMA (Preferred Reporting Items for Systematic Reviews and Meta-Analyses) guidelines, this study seeks to classify studies on analytical thinking based on year and country (Page et al., 2021; Rethlefsen et al., 2021). The PRISMA framework ensures a transparent, methodical approach to the literature review process, encompassing key steps such as defining research objectives, conducting extensive searches, evaluating and extracting data, assessing quality, analyzing findings, and presenting conclusions (Tricco et al., 2018). This rigorous approach not only provides a comprehensive understanding of the current state of research but also identifies gaps in knowledge and offers reliable evidence to inform decision-making. The keywords used during the article searching process include basic terms related to the topic of study as well as information related to the question of this study, such as “Analytical Thinking,” “Mathematical Thinking,” “Critical Thinking,” “Thinking” as well as other keywords as listed in Table 1.

**Table 1:** Keywords used for article search.

No.	Database	Keywords
1	WoS	TITLE-ABS-KEY ((“Analytical thinking” OR “Analy* thinking” OR “AT” OR “Critical thinking” OR “Critic* thinking” OR “CT”) AND (“Pupils” OR “Students”) AND (“Math” OR “Mathematics” OR “Mathematical” OR “Geometry” OR “Algorithm” OR “Calculus” OR “Statistics” OR “Function” OR “Equation”))
2	Scopus	TS = ((“Analytical thinking” OR “Analy* thinking” OR “AT” OR “Critical thinking” OR “Critic* thinking” OR “CT”) AND (“Pupils” OR “Students”) AND (“Math” OR “Mathematics” OR “Mathematical” OR “Geometry” OR “Algorithm” OR “Calculus” OR “Statistics” OR “Function” OR “Equation”))
3	Google Scholar	Analytical Thinking AND mathematic*

### 2.1 Searching strategy

Firstly, the selected databases were identified. Three databases such as Web of Science, Scopus and Google Scholar were selected (Masdoki and Din, 2021). These databases were chosen due to their extensive coverage of high-quality, peer-reviewed academic articles. They are well-regarded for their broad indexing of scholarly literature, ensuring a comprehensive and representative selection of relevant research on analytical thinking. Secondly, the search keywords were selected. To ensure that analytical thinking related articles were selected and other irrelevant literature was excluded, our search keywords were shown in Table 1 and the full text was searched. Finally, the research process was clarified. The whole study is divided into four steps, identification, screening, eligibility and inclusion as mentioned in PRISMA (Page et al., 2021; Rethlefsen et al., 2021). In the Identification phase, a comprehensive search strategy is developed by selecting appropriate databases (Web of Science, Scopus and Google Scholar), relevant keywords, and search filters to gather literature on analytical thinking in mathematics education. Screening involves a preliminary examination where irrelevant and duplicate references are removed. During the Eligibility phase, the remaining articles are assessed based on predefined inclusion criteria, such as accessibility, document type, language, and subject relevance, ensuring that only studies pertinent to the research questions are retained. Finally, in the Inclusion phase, the eligible studies are reviewed in full, and those meeting all criteria are included in the systematic review. This process adheres to established guidelines to maintain rigor, transparency, and consistency.

### 2.2 Selection criteria

**Figure 1:**
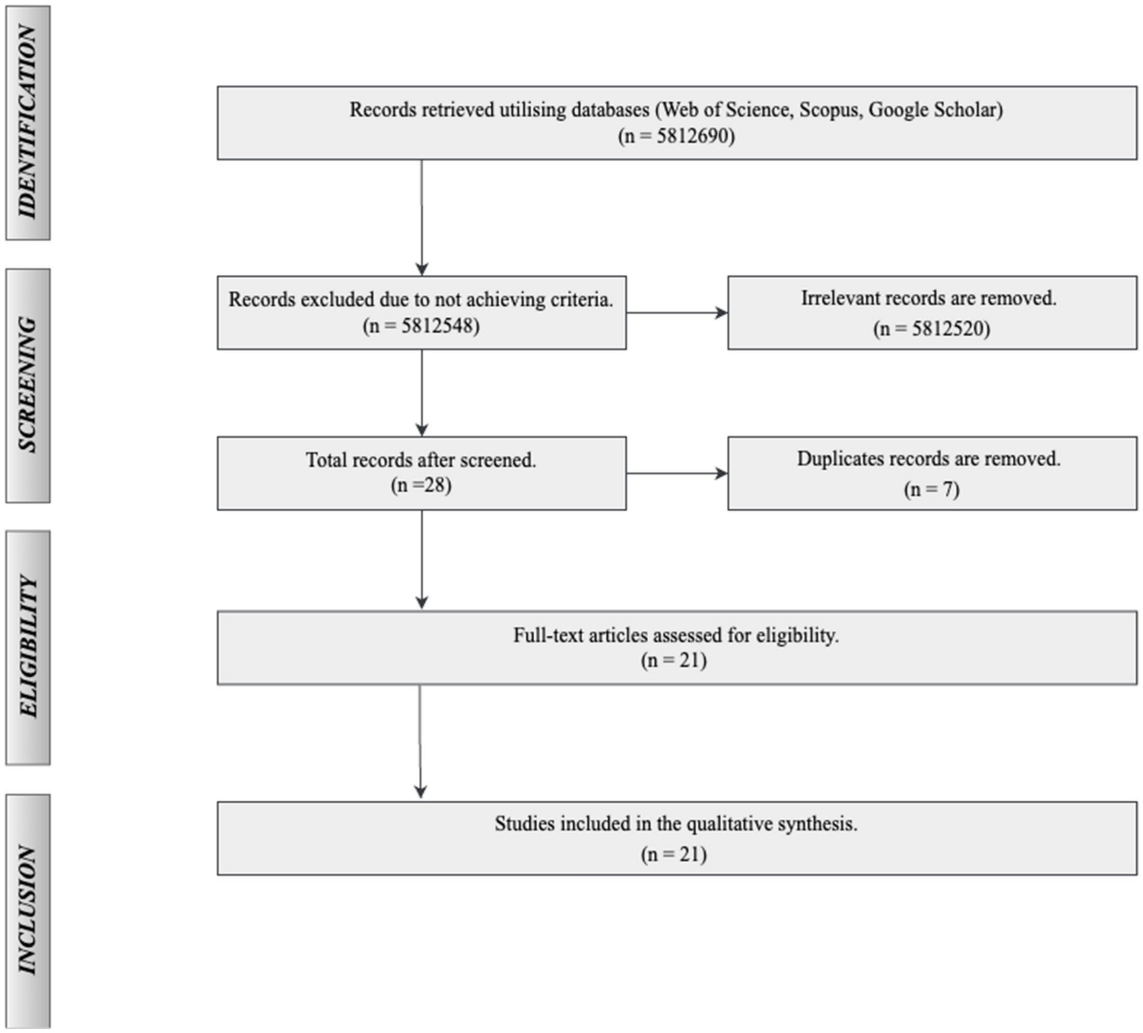
Article selection process.

In order to obtain articles that are sought after and appropriate, several stages of filters are used to filter the original articles. In the first step of the filtering stage, several acceptance and rejection criteria are used. The four criteria for rejection are: (1) the article is not fully accessible, (2) it is not a journal or conference article (e.g., a dissertation), (3) it is written in a language other than English, and (4) it is irrelevant to analytical thinking in mathematics. Four criteria for acceptance are: (1) the article is available, (2) it is a journal or conference article, (3) it is written in English, and (4) it is relevant to analytical thinking in mathematics. The second step in the screening phase was to remove duplicate and irrelevant articles by reading the titles and abstracts. The final analysis is done by a thorough and in-depth reading of the remaining articles in order to remove articles that are not relevant to the research needs (Brereton et al., 2007). The whole process of SLR can be seen in Figure 1, where 21 articles were finally selected as the final result of the search and filtering.

## 3 Results

This study adopts a SLR approach following the PRISMA guidelines to ensure a transparent and rigorous review process. The review focuses on classifying research related to analytical thinking in mathematics education by year and country. A total of 21 articles were included based on predefined inclusion and exclusion criteria, as outlined in Table 2. These articles were selected because they met the research objectives and passed the filtering process. Across the reviewed literature, common research methods include qualitative, quantitative, and mixed-method approaches. Many studies employed established theoretical frameworks, such as Bloom’s Taxonomy, critical thinking models, or analytical thinking process models. Frequently used assessment tools include structured observations, standardized tests, and task-based evaluations aimed at measuring students’ analytical thinking abilities.

**Table 2:** Analysis of articles eligible for acceptance and study within the scope of this study.

Author & year	Country	Title	Objectives
Wijaya et al. (2023)	Indonesia	How are students’ prior knowledge differentiate analytical thinking process in identifying the convergence of real number sequences?	This study explored students’ analytical thinking in determining real number sequence convergence, emphasizing the role of prior knowledge. It identified three cognitive processes and found a strong correlation between prior knowledge and analytical skills, highlighting its importance in mathematics education.
Abass and Al-Kinani (2022)	Iraq	Analytical thinking and its relationship to 21st century skills among secondary school females’ students in mathematics	This study investigated analytical thinking and 21st-century skills among female secondary students in Dujail, Iraq. Results from 295 participants indicated a positive correlation between these skills, highlighting the significance of analytical thinking in mathematics education.
Supriadi et al. (2022)	Indonesia	Implementation of a realistic mathematics learning approach (RME) and analytical thinking: The impact on students’ understanding of mathematical concepts in Indonesia	This study aims to determine the impact of a realistic mathematics learning approach (RME) and analytical thinking on students’ understanding of mathematical concepts.
Purba and Azis (2022)	Indonesia	The effectiveness of problem based learning model on the ability to solve mathematical problems in terms of students’ analytical thinking ability	This study aims to determine whether the PBL. Model effective in improving the ability to solve mathematical problems.
Mateus-Nieves and Díaz (2021)	Colombia	Development of mathematical thinking skill from the formulation and resolution of verbal arithmetic problems	Articulate the skills of mathematical thinking with the formulation and resolution of verbal statement arithmetic problems (PAVE).
Huincahue et al. (2021)	Germany	Mathematical thinking styles—the advantage of analytic thinkers when learning mathematics	This study explores the relationship between students’ mathematical thinking styles (MTS) and their mathematical performance. The study concludes that students who prefer analytical thinking tend to perform better in school, possibly due to the higher emphasis on analytical mathematical thinking in the evaluation process.
Anggoro et al. (2021)	Indonesia	Mathematical-analytical thinking skills: the impacts and interactions of open-ended learning method & self-awareness	This research aims to investigate various aspects of mathematical analytical thinking skills.
Suparman and Tamur (2021)	Indonesia	Problem-based learning for mathematical critical thinking skills: a meta-analysis	This study aims to evaluate, summarize, and estimate the PBL implementation for students’ MCTS during the last four years.
Syaiful et al., 2021	Indonesia	Problem-based learning model on mathematical analytical thinking ability and science process skills	This research examines the differences and relationships between students’ analytical thinking skills and science process skills with problem-based learning models in mathematics.
Setiana and Purwoko (2021)	Indonesia	The application of mathematics learning model to stimulate mathematical critical thinking skills of senior high school students.	The objective of this research is to analyze the twelfth graders’ mathematics critical thinking skills using a mathematics learning model to stimulate fundamental critical thinking abilities of science courses in SMA Negeri, Pacitan Regency, East Java Province, Indonesia.
Annizar et al. (2021)	Indonesia	The process of student analytical thinking in understanding and applying lattice method to solve mathematical problem	This research aims to describe the process of analytical thinking students in understanding and applying the Lattice Method to solve mathematical problems.
Kesorn et al. (2020)	Tailand	Development of an assessment tool for mathematical reading, analytical thinking and mathematical writing	The main objective of this research was to develop and validate the quality of an assessment tool for evaluating the mathematical reading, analytical thinking, and mathematical writing skills of fourth-grade students.
Faizah et al. (2020)	Indonesia	Exploring students’ thinking process in mathematical proof of abstract algebra based on Mason’s framework	A mathematical proof is a formal process which needs the ability of analytical thinking to solve. The aim of this research is to explore students’ thinking process in conducting mathematical proof based on Mason’s framework.
Reinke (2019)	America	Toward an analytical framework for contextual problem-based mathematics instruction	The author proposes an analytical framework, developed through observations of a contextual problem-based algebra unit.
Belecina and Ocampo (2018)	Philippine	Effecting change on students’ critical thinking in problem solving	This study examines the impact of problem situations on graduate students’ critical thinking in problem-solving within educational statistics, demonstrating significant improvement in analytical skills and suggesting the efficacy of this approach for enhancing mathematical reasoning across various domains.
Kadir (2017)	Indonesia	Meta-analysis of the effect of learning intervention toward mathematical thinking on research and publication of student	The purpose of this study was to analyze the impact of mathematics learning interventions on students’ mathematical thinking abilities. These abilities encompass various aspects such as connectivity, communication, representation, problem-solving, logical reasoning, critical thinking, creativity, analytical thinking, generalization, quantitative skills, and adaptability. The research conducted by students predominantly employed experimental methods with a mixed-method approach and classroom action research.
Osman et al. (2016)	Malaysia	Identifying pertinent elements of critical thinking and mathematical thinking used in civil engineering practice in relation to engineering education	This paper focuses on explaining an analytic process in identifying pertinent elements of critical thinking and mathematical thinking used in real-world civil engineering practice.
Godino et al. (2013)	Spanish	Synergy between visual and analytical languages in mathematical thinking	This paper explores the relationship between language, visual thinking, and analytical thinking in mathematical problem-solving using the “onto-semiotic approach” framework. It highlights how these elements cooperate during mathematical activities.
Sam and Yong (2006)	Malaysia	Promoting mathematical thinking in the Malaysian classroom: issues and challenges	This paper discusses the definition of mathematical thinking in the Malaysian context, reviews literature to assess its implementation in math classrooms, and highlights challenges faced by Malaysian math teachers in promoting mathematical thinking. It concludes with recommendations, such as effective lesson planning through Lesson Study collaboration, to foster mathematical thinking in classroom teaching.
Ferri (2003)	Germany	Mathematical thinking styles-an empirical study	In the study described in this paper, mathematical thinking styles of 15 and 16-year-old pupils shall be reconstructed.
Stein et al. (1996)	American	Building student capacity for mathematical thinking and reasoning: an analysis of mathematical tasks used in reform classrooms	This article focuses on the use of mathematical tasks as essential tools for nurturing students’ mathematical thinking and reasoning abilities.

For objective number one, the study spans from year 1996 to present, revealing an increasing trend in research as year progresses. For objective number two, the study identifies a focus on various countries including Indonesia, the United States, Thailand, Colombia, Pakistan, Malaysia, Germany, Spain, and Turkey, aiming to comprehend the international landscape of research on analytical thinking. Table 2 reports the analysis of the articles accepted and studied within the scope of this research, detailing the author, year, country, title, and objectives of each study.

### 3.1 The classification trend of studies on analytical thinking by year

Understanding the distribution of research on analytical thinking over year is crucial for identifying key developments and shifts in focus within the field. Analyzing trends by year provides insight into periods of heightened academic interest and the emergence of significant contributions. This section explores the chronological pattern of studies on analytical thinking, highlighting notable peaks and the overall growth trajectory of research in this area. By examining these trends, we can better understand the progression and current state of analytical thinking research across different year periods.

**Figure 2:**
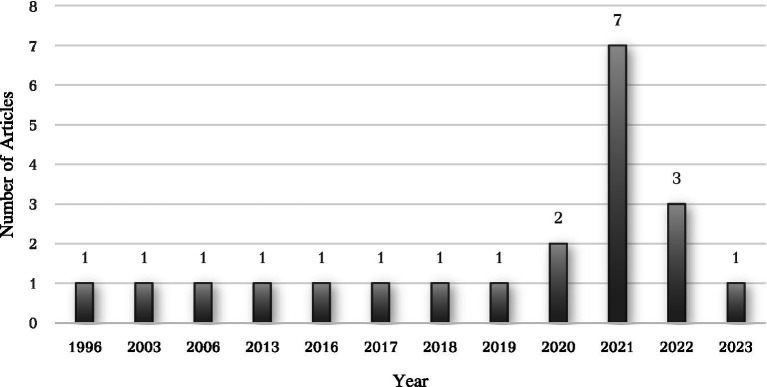
Years distribution of analytical thinking studies.

Figure 2 presents the distribution of data across different years from 1996 to 2023. The data shows that several years, such as 1996, 2003, 2006, 2013, 2016, 2017, 2018, and 2019, each have a frequency of 1, indicating relatively low data occurrences in those years. Notably, 2020 stands out with a frequency of 2. The years 2021- and 2022-mark significant peaks in the distribution, with 7 and 3 occurrences respectively, suggesting a concentration of data or research activity during this period. The frequency decreases slightly in 2022 and 2023, with 3 and 1 occurrences, respectively.

### 3.2 The classification trend of studies on analytical thinking by countries

Examining the country distribution of research on analytical thinking reveals how different countries contribute to the development of this field. By classifying studies based on country, we can identify regional research hubs, understand the global spread of interest in analytical thinking, and explore the role of local educational priorities in shaping research agendas. This section provides an overview of the countries leading in analytical thinking research, shedding light on patterns of international collaboration and regional focus in this growing area of study.

**Figure 3:**
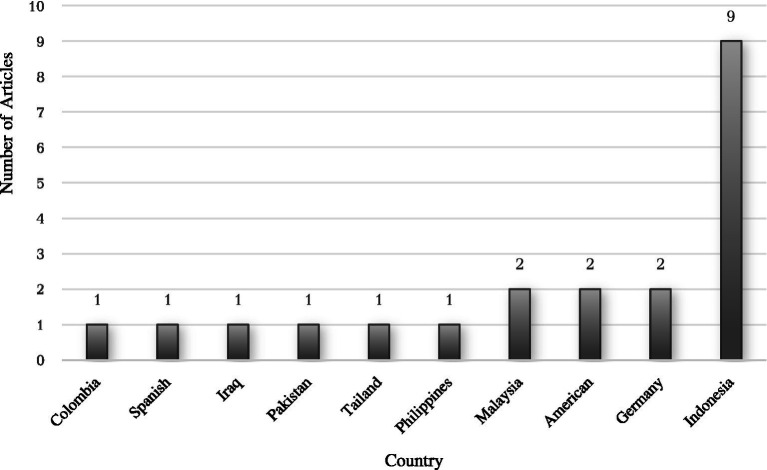
Countries distribution of analytical thinking studies.

Figure 3 illustrates the distribution of data across different countries. The chart reveals that the data is most heavily concentrated in Indonesia, which accounts for 9 occurrences, significantly higher than any other country. Malaysia follows with 2 occurrences, and both the United States and Germany have 2 occurrences each. Other countries, including Colombia, Spain, Iraq, Pakistan, Thailand, and the Philippines, each have a frequency of 1, indicating a more limited representation in the dataset.

## 4 Discussion

The growing prominence of analytical thinking in mathematics education is evidenced by recent trends observed in global research.

For the first objective, the increase in research activity after 2020 reflects a growing recognition of the need for analytical thinking to address the complexities of modern education, as highlighted by the systematic literature review methodology used in this study (Page et al., 2021). This trend underscores the pivotal role that analytical thinking plays not only in improving students’ cognitive abilities but also in fostering critical thinking and problem-solving skills essential for academic and professional success (Abrami et al., 2015). In mathematics education specifically, analytical thinking enables students to deconstruct mathematical problems, identify logical steps, and apply reasoning to reach solutions. Several studies included in this review applied interventions such as problem-based learning (Purba and Azis, 2022) and mathematical proof tasks (Faizah et al., 2020), which have shown to significantly enhance students’ analytical performance.

The fluctuation in research activity over year, particularly the surge in publications in 2021, can be attributed to several factors, including the impact of the COVID-19 pandemic and the growing recognition of the importance of analytical thinking in addressing complex educational challenges (Zhu and Liu, 2020). The pandemic led to a temporary decline in research productivity as institutions worldwide grappled with unprecedented disruptions. However, the subsequent rebound in 2021 and 2022 reflects a rapid adaptation to the new normal, where researchers increasingly focused on addressing the educational challenges exacerbated by the pandemic. This surge is consistent with the broader global trend of prioritizing research on analytical and critical thinking as essential skills in navigating complex, data-driven environments (Sarker, 2021). Additionally, the increased attention to these skills during and after the pandemic underscores the urgency of equipping students with the ability to think critically in uncertain times (Page et al., 2021).

For the second objective, factors related to countries have also played a crucial role in shaping the current landscape of analytical thinking research. In terms of countries distribution, Indonesia has emerged as a significant contributor to the body of research on analytical thinking, particularly in the field of mathematics education. Several factors contribute to this high output. Firstly, Indonesia’s educational policies have increasingly emphasized the importance of STEM (Science, Technology, Engineering, and Mathematics) education, which naturally fosters analytical thinking skills (Wijaya et al., 2023). Furthermore, Indonesia has made substantial investments in research and development, particularly in educational research, aiming to improve national educational standards and global competitiveness. This emphasis on research is also supported by international collaborations and a growing number of research institutions focused on educational innovation. Additionally, the cultural context in Indonesia, which values educational attainment and intellectual development, further encourages scholarly activity in this area. These factors combined have positioned Indonesia as a leading nation in contributing to the global discourse on analytical thinking in education (Huincahue et al., 2021). Thus, both year and country factors have played crucial roles in shaping the current landscape of research on analytical thinking.

The articles chosen for this systematic literature review highlight the critical role of analytical thinking in enhancing cognitive processes and problem-solving abilities in educational settings, making a significant contribution to the theoretical understanding of analytical thinking. Beyond theoretical contributions, several studies also proposed practical frameworks for classroom implementation. For example, instructional strategies such as open-ended problem solving and task-based assessments were commonly used to cultivate analytical thinking in mathematics (Anggoro et al., 2021; Kesorn et al., 2020).

Recent research has emphasized the necessity of developing analytical thinking skills as foundational for academic success and lifelong learning (Cromwell et al., 2023). However, instead of reiterating this conclusion, it is important to highlight what works well and why. For instance, task-based assessments proved more effective than standardized tests in evaluating students’ reasoning processes (Kesorn et al., 2020). Conversely, some studies noted difficulties in implementing abstract proof-based thinking tasks due to students’ prior conceptual gaps (Faizah et al., 2020).

Moreover, the practical implications of these findings are far-reaching, as they provide valuable insights for educators, policymakers, and practitioners aiming to foster analytical skills in students. By integrating analytical thinking into curricula, educational systems can better prepare students to navigate complex challenges in both academic and real-world contexts (Almulla and Al-Rahmi, 2023). These insights align with the growing recognition of the importance of analytical and critical thinking in the 21st century, particularly in STEM education (AlAli, 2024).

To enhance future efforts, it is recommended that teacher training programs emphasize how analytical thinking can be nurtured through specific math tasks, such as proof construction, data modeling, and algebraic reasoning. Likewise, curriculum designers should consider integrating interdisciplinary elements and educational technologies that support analytical skill development across diverse cultural contexts.

Analytical thinking is a cornerstone of mathematical learning, and it plays an important role in developing students’ deep thinking, problem-solving, and critical thinking skills. Therefore, educational practice can further draw on the findings of analytical thinking research to design more effective teaching strategies and curricula to promote students’ academic performance and thinking development. This provides an important reference point for educational reform and innovation in teaching methods.

## 5 Conclusion

The purpose of this study is to systematically review and summarize the trends in the field of mathematical analytical thinking, specifically focusing on year and country patterns. Analytical thinking, which involves deeply understanding and dissecting information, is crucial in today’s data-rich society. In education, it enhances students’ ability to think deeply, deconstruct problems, and evaluate evidence, thereby improving academic performance and critical thinking skills. However, previous research has not thoroughly examined the research patterns of analytical thinking, particularly in terms of year and country classifications. This study classified relevant studies by year and country to better understand these trends. It can be concluded that the observed fluctuation in research activity, with a sharp increase in 2020, 2021, and 2022, can be attributed to the accumulation of articles during the COVID-19 pandemic, which led to a surge in studies. The study also identifies Indonesia as leading contributors to this body of research, indicating a global interest in developing these essential cognitive abilities. These insights help fill the gap in understanding the patterns and trends of analytical thinking research, particularly within the context of mathematics education.

Acknowledging the limitations of this study is crucial for understanding the context in which the findings should be interpreted. One significant limitation is the reliance on data sources that may not fully capture the diversity of research on analytical thinking across different educational systems. For instance, much of the data was drawn from databases that predominantly include English-language publications, potentially overlooking relevant studies published in other languages. Additionally, the research on mathematical analytical thinking is relatively limited and often embedded within broader studies, requiring careful extraction and analysis. This process introduces a degree of subjectivity, as the selection of relevant material may be influenced by the researchers’ interpretations. These limitations suggest that the findings should be interpreted with caution, particularly when generalizing to non-English speaking contexts or when considering the broader scope of analytical thinking research.

Future research should focus on addressing the current limitations by incorporating more diverse data sources and broadening the scope to include studies published in non-English languages. Expanding and refining the theoretical framework of analytical thinking is essential, particularly by investigating its application across various disciplines and fields. Interdisciplinary research will be pivotal in enhancing our understanding of analytical thinking’s impact and fostering innovation in its practical applications. Additionally, the development of a mathematical analytical thinking questionnaire, incorporating psychological factors and using the Rasch model for validation, will be crucial for more precise and reliable measurement tools in this field (Kamaruddin and Mohd Matore, 2021; Mohd Noh and Mohd Matore, 2022; Sovey et al., 2022). By concentrating on these areas, this finding has important implications to refine the implementation of analytical thinking in both academic and educational contexts, thereby contributing to the continued development and advancement of related fields. Ongoing research and efforts will enable a more comprehensive understanding and effective integration of analytical thinking, ultimately improving educational practices and outcomes.

In conclusion, the cultivation of analytical thinking is crucial for preparing students with the skills needed to navigate the complexities of modern society. As educational systems evolve, it is imperative to prioritize analytical thinking as a fundamental component of the curriculum. This focus will not only boost academic performance but also equip students to meet the demands of an increasingly data-driven world. Continued research in this area is essential for advancing educational practices and ensuring that students are adequately prepared for the future.

## Data Availability

The original contributions presented in the study are included in the article/supplementary material, further inquiries can be directed to the corresponding author.
